# Aquatic Biosystems reviewer acknowledgement 2013

**DOI:** 10.1186/2046-9063-10-1

**Published:** 2014-01-16

**Authors:** Edward J Phlips, Shiladitya DasSarma

**Affiliations:** 1 Department of Fisheries and Aquatic Sciences, University of Florida, 7922 NW 71st Street, Gainsville, FL 32653, USA; 2 Department of Microbiology and Immunology, Institute of Marine and Environmental Technology, University of Maryland, 701 East Pratt Street, Baltimore, MD 21202, USA

## Abstract

**Contributing reviewers:**

The *Aquatic Biosystems* editorial team would like to thank the following colleagues who contributed to peer review for the journal in 2013.

## 

Javier Alcocer

Mexico

Kartik Baruah

Belgium

Zenon Batang

Saudi Arabia

Donald Behringer

United States of America

Peter Casper

Germany

Feng Chen

United States of America

Diógenes Felix da Silva Costa

Portugal

David Gauthier

United States of America

Baban Ingole

India

Luke Iwanowicz

United States of America

Jong-Myoung Kim

Korea, South

Miroslav Macek

Mexico

Ramon Massana

Spain

Maria Rosa Miracle

Spain

Uwe Münster

Finland

Rufus Kitto Muthunayagam

Saudi Arabia

Christopher Ottinger

United States of America

Wolf Pecher

United States of America

Danielle Puls

United States of America

Nico Salmaso

Italy

Terry Snell

United States of America

Luis Soto

Mexico

Evan Stephens

Australia

Gabriel Strain

United States of America

Sherry Tamone

United States of America

Amandine Vaslet

France

Andrea Waringer-Löschenkohl

Austria

Yik Sung Yeong

Malaysia

